# ApoE4: an emerging therapeutic target for Alzheimer’s disease

**DOI:** 10.1186/s12916-019-1299-4

**Published:** 2019-03-20

**Authors:** Mirna Safieh, Amos D. Korczyn, Daniel M. Michaelson

**Affiliations:** 1 Department of Neurobiology, Sagol School of Neurosciences, The George S. Wise Faculty of Life Sciences, Tel Aviv University, Ramat Aviv, 6997801 Tel Aviv, Israel; 2 Departments of Neurology and Pharmacology, Sackler Faculty of Medicine, Tel Aviv University, Tel Aviv, Israel

**Keywords:** Alzheimer’s disease, Apolipoprotein E4, apoE4 therapeutics, apoE4 lipidation, Anti-apoE4 antibodies, Human apoE-targeted replacement mice

## Abstract

**Background:**

The growing body of evidence indicating the heterogeneity of Alzheimer’s disease (AD), coupled with disappointing clinical studies directed at a fit-for-all therapy, suggest that the development of a single magic cure suitable for all cases may not be possible. This calls for a shift in paradigm where targeted treatment is developed for specific AD subpopulations that share distinct genetic or pathological properties. Apolipoprotein E4 (apoE4), the most prevalent genetic risk factor of AD, is expressed in more than half of AD patients and is thus an important possible AD therapeutic target.

**Review:**

This review focuses initially on the pathological effects of apoE4 in AD, as well as on the corresponding cellular and animal models and the suggested cellular and molecular mechanisms which mediate them. The second part of the review focuses on recent apoE4-targeted (from the *APOE* gene to the apoE protein and its interactors) therapeutic approaches that have been developed in animal models and are ready to be translated to human. Further, the issue of whether the pathological effects of apoE4 are due to loss of protective function or due to gain of toxic function is discussed herein. It is possible that both mechanisms coexist, with certain constituents of the apoE4 molecule and/or its downstream signaling mediating a toxic effect, while others are associated with a loss of protective function.

**Conclusion:**

ApoE4 is a promising AD therapeutic target that remains understudied. Recent studies are now paving the way for effective apoE4-directed AD treatment approaches.

## Background

Senile dementia is one of the greatest medical threats of the twenty-first century. Thus, unsurprisingly, considerable intellectual and financial resources have been invested to forestall this emerging disaster. Unfortunately, to date, these efforts are yet to succeed in identifying a viable solution.

Many disorders of brain function lead to cognitive decline, among which Alzheimer’s disease (AD) is considered the most prevalent. The definition of AD itself is not simple. Unique among human diseases, the accepted definition requires a combination of clinical manifestations (i.e., dementia) and structural changes, namely deposition of amyloid β (Aβ) and hyperphosphorylated tau tangles (neurofibrillary tangles) in the brain. Since various other brain disorders can lead to cognitive deterioration comparable to that observed in AD and, similarly, identical pathological changes can occur in people who do not manifest cognitive decline, diagnoses of AD require both specific cognitive deterioration and neuropathological changes. It is therefore unsurprising that attempts to focus treatment on deposits have so far resulted in disappointment [1, 2].

The term AD has itself undergone changes in definition. For approximately 50 years, since its first description, AD was specifically used to describe the development of dementia in younger people who had Aβ plaques and neurofibrillary tangle deposits; presently, this is termed early-onset AD (EOAD). However, as has been discovered over the years, EOAD results primarily from genetic mutations in certain genes. Nevertheless, current demographics being diagnosed with AD are older people without any of these mutations.

Identification of the specific EOAD mutations enabled the creation of animal models of the disease, with transgenic mouse models revolutionizing AD research and the development of experimental therapies. Nevertheless, it must be stressed that these models are specific for EOAD [3] and not for the much more common, late-onset form of the disease (LOAD), despite them having been employed for this. Therefore, given the different factors that play a role in both forms of the disease, these approaches have important limitations in their application to LOAD. In addition, the behavior phenotype of mouse models differs substantially from that of human disease. The cognitive deficits of AD mice are rather mild when compared with those of dementia – whereas humans with AD eventually become helpless and require constant care, the mice remain independent until their death; further, comorbidities, inflammation, and neural loss are less pronounced.

Epidemiologic studies exploring the risk factors for LOAD elucidated a large number of associated factors, including important vascular processes [4, 5]. Additionally, a number of genetic associations have been revealed by genome-wide association studies [6], the most important of which is the apolipoprotein E gene on chromosome 17 (*APOE* gene, apoE protein) [7]. Other polymorphisms are associated with genes related to inflammation and immune responses, lipid metabolism, and endocytosis/intracellular trafficking [8], but none of them are as common nor do they have an effect as strong as that of apoE. To date, attempts to modify the molecular processes involved in AD have mainly targeted Aβ and, more recently, tau [1, 9]; nevertheless, these attempts have been mostly unsuccessful. Herein, we discuss another possible, albeit less popular target – apoE.

### Role of apoE

Several studies have demonstrated the important involvement of apoE in AD. This was first suggested by Strittmatter and Roses [10], who showed that, of the three polymorphic forms of *APOE*, namely *APOE2*, *APOE3*, and *APOE4*, carriers of *APOE4* are more likely to develop AD. Further, the cognitive changes in the *APOE*4 carriers were shown to occur several years earlier, with a dose-dependent effect. Conversely, *APOE2* carriers have a ‘protective’ effect relative to *APOE3* and *APOE4* carriers, and therefore the apoE4 protein appears to be ‘toxic’, whereas apoE2 is ‘protective’ against AD. This assumption will be discussed critically herein since, theoretically, all isoforms could be ‘protective’, with apoE2 having the strongest and apoE4 the mildest effect or, vice versa, all isoforms may have ‘toxic’ features of varying degrees. It is therefore likely that apoE has several effects, some of which are protective whereas others are toxic, and that apoE4 has the least beneficial expression of these features. This may have important implications, since knowing the dominant effect of apoE4, and whether it is toxic or protective, would affect the therapeutic strategy used to treat apoE-related disease. Importantly, apoE4 has been implicated in numerous processes, including crosstalk with Aβ, and shown to have an effect on lipid metabolism and inflammation [11–13]; however, the relative importance of these processes in mediating the effect of apoE4 in AD remains to be determined.

Another issue of concern is that the serum, cerebrospinal fluid (CSF), and presumably tissue concentrations of the different haplotypes are not equal; carriers of *APOE4* have lower serum and brain apoE concentrations than do carriers of the other isoforms [14], and it is possible that some of the apoE effects depend on the apoE concentration rather than its quality. Considering the simplest assumption first, namely that apoE4 is toxic to the brain, may suggest that blocking its action may delay or stop the development of AD. Blocking the apoE4 effect specifically can be achieved by genetic, biochemical, and immunological methods. Such an approach would help the 40–60% of AD patients who carry apoE4, whereas, if all apoE forms are in fact toxic (albeit to a different degree), a better approach would be to block all apoE action, at least in the adult brain, if this can be done with impunity.

Additionally, it is important to consider that, although the apoE protein is synthesized primary in the liver, it is also produced in the brain and functions there in many in many capacities, some of which may be relevant to AD. One of the major roles of apoE in the brain, similar to the rest of the body, is related to lipid transport and cholesterol homeostasis [15–17]. ApoE4 was shown to be hypolipidated and less effective than apoE3 in inducing cholesterol efflux, suggesting that the pathological effects of apoE4 are related to lipid metabolism. This assertion and other mechanistic studies, such as the role of interaction of apoE4 with Aβ, phosphorylation of tau protein, disruption of metachondrial function, and others discussed in this review, have been studied extensively experimentally, both in whole animals and in isolated tissues in vitro. However, thus far, these studies have not identified one function that can be regarded as the single most likely and important pathway. Nevertheless, the limitations of these experimental methods need to be examined critically. In particular, it should be stressed that none of the available models can be fully considered to be representative models of AD as a complex disease. Furthermore, the levels of expression of Aβ and tau in AD models are often non-physiological, rendering it difficult to assess the significance of downstream signaling effects. In addition, endogenous rodent molecules may react differently with human AD molecules than do their human counterparts [18]. It is important to also note that not all the genes that have been linked to apoE4 and AD (e.g., *TOMM40*, which is situated very close to the *APOE* gene on chromosome 19 and whose different isoforms are closely linked to *APOE* alleles [19]) have been studied at the animal model level.

### Impact of the *APOE* genotype on other diseases

Numerous studies, backed by meta-analyses, have revealed that *APOE4* is also a risk factor for other diseases [20], including cerebral amyloid angiopathy (CAA) [21], dementia with Lewy bodies (DLB) [22], tauopathy [23], cerebrovascular disease [24], multiple sclerosis [25, 26], and vascular dementia [24, 27], as well as being related to poor outcome following head injury [28, 29]. However, the involvement of *APOE2* in these diseases is less clear, presumably in part due to the low abundance of *APOE2* carriers in the population. The *APOE* genotype also plays a role in age-related macular degeneration (AMD), where paradoxically *APOE4* is protective [30]. Pathologically, AMD is associated with excessive angiogenesis and is being treated by anti-vascular endothelial growth factor (VEGF) antibodies that reduce excessive pathological angiogenesis [31]. In contrast, AD and other diseases for which apoE4 is a risk factor are characterized by enhanced degeneration and impaired plastic repair [32, 33]. Animal and cellular model studies revealed that APOE4 is associated with impaired cellular plasticity [32, 34, 35]. It is thus likely that the negative effects of APOE4 in AD are due to this impaired neuronal synaptic plasticity, whereas in AMD, in which the key pathology is increased angiogenesis and vascular plasticity, the effects of apoE4 could be protective due to the reduction in retinal pathological neovascularization [36].

Regarding the association of *APOE4* with DLB risk, it has been shown that *APOE4* is a strong risk factor across the DLB spectrum, being associated with an increased likelihood of presenting with dementia in the cortex of a pure synucleinopathy [22]. Accumulating data suggest that this effect of APOE4 on the pathology of DLB is through a non-amyloid-related mechanism, which merits further investigation [22]. Further, it has been recently shown that APOE4 markedly exacerbates tau-mediated neurodegeneration in a mouse model of tauopathy [23]; this finding, and the observation that tauopathy associated with frontotemporal dementia is associated with increased apoE4 allele frequency, suggest that the involvement of apoE4 in tauopathy may be independent of Aβ [37, 38].

Epidemiological studies have consistently shown that AD patients have an increased load of cerebrovascular diseases [39]. Since apoE is a carrier of plasma cholesterol, it is of interest to determine the contribution of cardiovascular disease pathology to dementia in carriers of different *APOE* isoforms. Apparently, *APOE4* carriers are more likely to develop ischemic cardiovascular diseases (OR 1.68, 95% CI 1.36–2.09) [40, 41]. Moreover, an autopsy-based study established that *APOE4* is a significant risk factor for cerebral ischemia, with a three-fold increase over *APOE3* [42]; yet the effect is relatively small and has not been confirmed in other studies [43, 44]. Additionally, accumulating data have confirmed an association between *APOE4* and cerebral microbleeds, which may be due to the effect of apoE4 on amyloid deposition around leptomeningeal vessels [45]. Nevertheless, most of these results were based on clinical observations, which are subject to referral bias and diagnostic inaccuracies.

Since *APOE4* is the most prevalent genetic risk factor for AD, it is not surprising that the *APOE* genotype has been reported to affect the outcome of clinical trials directed at different therapeutic targets [46]. These findings are likely due to indirect effects where the target of the therapeutic treatment interacts with apoE4. In this review, we address the therapeutic potential of treatments by focusing on the *APOE4* gene and apoE4 protein as well as on key downstream targets of apoE4.

## Review of suggested apoE-driven mechanisms

The presentation of AD as well as animal and cellular studies led to the generation of several, not mutually exclusive, hypotheses regarding the cellular and molecular mechanisms that may mediate the pathological effects of apoE4. The following section summarizes the main mechanisms by which apoE may be involved in AD.

### Aβ metabolism

Aβ deposition in AD patients is more abundant in apoE4 carriers in comparison with non-carriers [47]. Moreover, similar results were observed even in cognitively normal elderly subjects (although this association was weaker than seen in demented individuals) [48–50]. As mentioned above, lower CSF and plasma concentrations of apoE are found in *APOE4* carriers, suggesting that lower levels of apoE might facilitate the accumulation of Aβ in the brain; this was supported by the finding that apoE levels are negatively correlated with Aβ levels in multiple brain regions when analyzed in non-demented individuals [51]. In addition, animal model studies utilizing targeted replacement mice that express human apoE4 or apoE3 and corresponding in vitro studies revealed that apoE4 affects several key steps in the amyloid cascade, including the aggregation and deposition of Aβ, which, like in humans, has the isoform dependency of apoE4 > apoE3 > apoE2, and Aβ clearance from the brain, which follows the opposite trend [52, 53]. It has been shown that apoE binds to Aβ and that blocking this binding with a 12–28 fragment of Aβ counteracts key in vivo and in vitro pathological effects of Aβ [54]. All together, these observations suggest that apoE4 may have specific brain area effects in regulating Aβ accumulation and may therefore play a key role in AD pathogenesis.

Accordingly, apoE4 enhances Aβ production by affecting the activity of gama-secretase [55]. With regards to clearance, apoE4 impairs the lysosomal degradation of Aβ, and it is less effective than apoE3 in transporting Aβ across the blood–brain barrier (BBB). Additionally, apoE4 has an impaired ability to facilitate the proteolytic degradation of Aβ by neprilysin and the insulin-degrading enzyme [56–59]. Finally, it has been shown that apoE4 likely promotes Aβ aggregation and stabilizes the Aβ oligomers to a greater degree than apoE3, and that it inhibits the conversion of oligomers into Aβ fibrils through the formation of apoE/Aβ complexes [48, 60].

For more detailed information regarding the interaction of Aβ and apoE, see previous reviews [12, 48, 59, 61–65].

### Tau phosphorylation

Hyperphosphorylated tau is a major constituent of neurofibrillary tangles. Analysis of CSF samples from AD patients and healthy controls revealed that the ratio between phosphorylated and total tau might serve as a biomarker of AD [32, 66–68]. Complementary animal models suggest that tau hyperphosphorylation alone can cause neurodegeneration, leading researchers to conclude that hyperphosphorylated tau is toxic to neurons, and suggesting that hyperphosphorylated tau plays a major role in AD neuropathology [66]. This has been observed in several mouse models, including in apoE4-targeted replacement mice in which most of the apoE is synthesized by astrocytes [69, 70] as well as in transgenic mice in which over-expression of apoE4 is under the neuronal promoter [71, 72]. Furthermore, tau hyperphosphorylation is enhanced following exposure to stress or injury [73]. Corresponding results have been demonstrated in cell cultures [74].

Two complementary mechanisms have been proposed to explain the effects of apoE4 on tau hyperphosphorylation. Firstly, a direct mechanism based on the fact that apoE3 is more effective in binding to non-phosphorylated tau than apoE4, thereby preventing tau accumulation. Secondly, it has been proposed that apoE4 in neurons can escape the secretory pathway [75] due to its unique structure and that it interacts directly with tau in the cytoplasm to induce its hyperphosphorylation [76]; this proposed indirect mechanism may be mediated by apoE receptor-driven signaling cascades specific to apoE4, which in turn modify the function of tau kinases and phosphatases [77]. In addition, the enhanced ability of apoE4 to escape the secretory pathway enables it to intracellularly interact with zinc to phosphorylate tau protein through erk activation [78].

### Transactive response DNA-binding protein 43 (TDP-43)

TDP-43, an RNA-binding protein that functions in axon skipping, has recently been shown to be deposited in AD brain. TDP-43 is present in the brain of 65–80% of AD patients and was shown to be associated with progressive hippocampal atrophy. Research investigating the cross-sectional association between apoE4 and TDP-43 by mapping the potential associations between apoE4 and tau, Aβ, and TDP-43, indicates that the deposits of this protein are also increased in *APOE4* carriers in comparison to *APOE3* and *APOE2* carriers [79–81].

### Lipid metabolism

ApoE, which is the brain’s most prevalent lipoprotein, is associated with cholesterol and phospholipids as high-density lipoprotein-like particles that play a key role in the distribution and recycling of lipids in the brain [17]. This led to extensive investigations of the possibility that lipids play an important role in mediating the pathological effects of apoE4. Measurement of the brain and CSF levels of docosahexaenoic acid (DHA), an essential ω-3 fatty acid critical for neuronal and brain function [82], revealed that DHA levels are reduced in AD patients [83] and in apoE4 carriers [84], and that apoE4 increases the uptake and incorporation of DHA into distinct brain areas [85]. Similar results were observed in apoE4-expressing mice [86], wherein the brain’s pathological effects of apoE4 were counteracted by feeding mice with a fish-oil high-DHA diet [84, 87]. Further studies revealed that apoE4 is associated with disruption of the BBB [88] and with phospholipid and cholesterol dysregulation [63, 89, 90].

The important role of cholesterol in a variety of cellular mechanisms and its pronounced effects on Aβ levels [91] and related mechanisms suggest that cholesterol is an important player in the pathogenesis of AD [92, 93]. It was reported that subjects with both an apoE4 genotype and high cholesterol levels have more pronounced cognitive decline than subjects expressing only one of these risk factors [94]; however, such effects were not seen in other human studies [95]. Mouse model studies revealed that a high cholesterol diet accentuates the pathological effects of apoE4 in targeted replacement mice that express human apoE isoforms and no mouse apoE [87]. It is important to note that, although these studies suggest a link between apoE4 and lipids, they do not provide a clear mechanism nor a therapeutic target.

Analysis of the degree of lipidation of the different apoE isoforms in human CSF and in the brains of apoE-targeted replacement mice revealed that, in both human and mice, apoE4 is hypolipidated relative to apoE3, and that brain apoE2 is the most lipidated isoform [96, 97]. The CSF apoE4 high-density lipoprotein-like particles [17] are smaller and less lipidated in apoE4 than in apoE3 carriers [98, 99]. The lipidation of apoE in the brain is driven by the ATP binding cassette proteins ABCA1 and ABCG1, wherein the former drives the initial lipidation of apoE, which is then further lipidated by ABCG1 [100]. Downregulation and deletion of ABCA1 reduce the levels of plasma and brain apoE and are associated with the formation of smaller apoE-containing lipoprotein particles [17, 101] and with the accentuation of the apoE4 phenotype [102]. This led to assessing the possibility that the pathological effects of apoE4 may be related to its hypolipidation and that the lipidating protein ABCA1 may be a promising therapeutic target [103, 104]. Evidence supporting this assertion is presented below (see *Approaches directed at the apoE4 protein*).

### Mitochondrial function

Extensive research has provided evidence that metabolic alterations resulting from mitochondrial dysfunction occur in AD [105] and are accentuated in *APOE4* carriers. Accordingly, gene expression studies revealed that apoE4 expression in AD, when compared to apoE3, is associated with downregulation of gene transcripts of mitochondrial respiratory complexes I, IV, and V [106, 107], in addition to an isoform-specific effect on the expression of oxidative stress and mitochondrial-related transport proteins [108]. These findings are in agreement with the fact that *APOE4* carriers develop AD-like cerebral glucose hypo-metabolism decades before the onset of the clinical features of AD [106, 107]. In vitro studies revealed that apoE4-driven mitochondrial dysfunction is related to its isoform-specific binding to the F1 mitochondrial ATP synthase [108], as well as to an impaired ability to control the levels of reactive oxygen species and interactions with cytoskeletal proteins [70, 109–111].

### Neuroinflammation

The association of activated microglia [112] and complement proteins [113] with brain AD lesions, as well the discovery that rheumatoid arthritis patients who were treated regularly with anti-inflammatory drugs are relatively spared from AD, led to the proposition that neuroinflammation plays a role in the pathogenesis of AD [114]. This association between AD and neuroinflammation is further supported by recent genome-wide association studies that showed a marked association between AD and distinct immunity-associated genes such as *CLU* and *TREM2* [115, 116]. Importantly, neuroinflammation is more pronounced in *APOE4* carriers [117–119] and in corresponding animal model studies, including co-localization of apoE with microglia in the brain [120, 121], suggesting a role for apoE in the innate immune response in AD brain. This is corroborated by the finding that, in mice, following inflammatory stimulation, *APOE4* carriers have an enhanced and prolonged neuroinflammatory response [47, 122–124]. This inflammation may be driven by the effects of apoE4 on microglial activation [60, 125] as well as by enhancing the levels of proinflammatory cytokines [123, 126]. Alternatively, it has been suggested that the inflammatory effects of apoE4 may be related to miRNA146a, which is the primary miRNA in the brain. This suggestion stems from the finding that the levels of miRNA146a are higher in brains of AD patients than in the corresponding mouse model. It is suggested that elevated miRNA146a levels lead to an insufficient negative feedback regulation of inflammation, resulting in chronic inflammation [127, 128], yet the apoE isotype-specific effects remain poorly understood. However, in view of the uncertainty as to when in the course of the disease neuroinflammation is beneficial or toxic, the timing and choice of inflammatory molecule to be targeted for the treatment of AD and apoE4-related inflammation remain to be determined. Indeed, this issue may be the underlying cause for the lack of effectiveness of prospective nonsteroidal anti-inflammatory drug (NSAID) treatments [129]. A meta-analysis of numerous studies revealed no beneficial effect of NSAIDs on cognition and overall severity of AD [129]. Nevertheless, recent epidemiological data suggest that *APOE4* carriers are better responders to NSAID treatment [61, 62, 89]. The mechanisms underlying this effect are not fully understood and may be related to the higher susceptibility of *APOE4* carriers to inflammation and oxidative stress [130].

Nevertheless, it is clear that AD inflammation-related studies should be stratified according to the *APOE* genotype.

### Vascular integrity/function

ApoE functions as a ligand for low-density lipoprotein (LDL) receptors and plays a role in lipid metabolism; it has been well described in the context of cardiovascular diseases [17, 131, 132]. Observations in AD brains using autopsy and imaging indicate cerebrovascular dysfunction, including disruption of microvascular integrity and reduced cerebral blood flow in addition to small vessel arteriosclerosis and amyloid angiopathy. These cerebrovascular changes are greater in *APOE4* carriers when compared to non-carriers [133–135].

Several mechanisms have been proposed regarding the effects of apoE4 on cerebrovascular integrity, one of which is related to the accumulation of Aβ in the AD cerebral vasculature, a condition known as CAA. The prevalence of CAA is elevated in *APOE4* carriers [21] and can severely disrupt the integrity of blood vessels, leading to hemodynamic disturbances and thrombosis as well as BBB dysfunction and microbleeds. Additionally, it has also been strongly associated with cognitive impairment in humans [136, 137].

Another molecule involved in vascular changes associated with AD pathophysiology and which is affected by apoE4 is fibrinogen. Both fibrinogen and fibrin accumulate in the AD neurovasculature [138], and through their interaction with Aβ, they lead to abnormality in fibrin clot formation, leading to a clot structure that is more resistant to enzymes responsible for degradation [139]. The accumulation of fibrin and fibrinogen along the vessel wall and in the tunica media is apoE isoform dependent (apoE4 > apoE3) [140]. The mechanisms underlying this outcome merit further investigation.

Other than these direct effects of apoE on the vascular integrity, it is important to note that the apoE isoforms also affect the efficiency of the efflux of Aβ across the BBB [141]. Accordingly, apoE4 disrupts the clearance of Aβ through the BBB by shifting the efflux from fast LDL receptor-related protein 1 (LRP1)-dependent transcytosis to slow very low-density LDL receptor (VLDLR)-dependent transcytosis [141], resulting in poor clearance of Aβ from the brain (which could explain the lower concentration of apoE in the CSF of *APOE4* carriers).

### Insulin and VEGF signaling

Recent advances suggest that both the insulin and the VEGF cascades are impaired in AD and are specifically affected by apoE4. Human and animal model studies revealed that AD is associated with reduced insulin levels in the CSF and with insulin resistance [142], as well as with lower levels of the insulin receptor substrate IRS1 and higher levels of p-IRS1, which is a marker of brain insulin resistance [143–145]. Examination of the APOE genotype specificity of these effects revealed that brain insulin metabolism in AD is differentially affected by the various apoE isoforms [146], and that apoE4 impairs neuronal insulin signaling and insulin receptor trafficking in corresponding cellular and animal models [147–150]. Clinical trials of AD and mild cognitive impairment patients utilizing intranasal and other modes of insulin administration revealed general improvements in cognitive functions such as memory and attention [142, 151, 152]. Examination of the APOE genotype specificity of this effect revealed that this treatment was most effective in *APOE4* carriers [153], although the lack of such specificity has also been reported [154].

VEGF, originally described as a key angiogenic factor, has recently been shown to play an important role in neurogenesis and neuroprotection and to affect neuronal plasticity and repair [155]. AD is associated with low serum VEGF levels [156], which are in turn associated with progressive loss of cognitive function [157]. Specific interactions between VEGF and apoE4 have been reported in both AD and mild cognitive impairment [158]. Animal model studies revealed that brain levels of VEGF and its receptor (VEGFR-2) were reduced in the hippocampus of apoE4-targeted replacement mice compared with the corresponding apoE3 mice and that upregulation of the levels of hippocampal VEGF utilizing a viral vector reversed the apoE4-driven accumulation of Aβ and hyperphosphorylated tau in hippocampal neurons and the associated synaptic and cognitive impairments [69].

### Synaptic plasticity

Finally, another feature of AD that is probably linked very significantly to memory impairment and cognitive decline is synaptic failure. ApoE isoforms differentially regulate synaptic plasticity and repair. Clinical studies suggest that *APOE4* carriers have lower levels of dendritic spine density in the hippocampus [159, 160], a finding that correlates well with the fact that apoE4 mice also have lower dendritic spine density and length compared with apoE3 mice [161–163] and suggests a different neuroprotective function of the isoforms. It is interesting to note that studies with 1-month-old mice revealed similar results, suggesting an early onset of apoE4-driven alteration of neuronal circuitry [164]. One of the key processes affected by apoE that leads to deficient synaptic plasticity is neurite (axon or dendritic) outgrowth. A large body of evidence has demonstrated that the apoE3 isoform promotes neurite outgrowth more effectively than apoE4, with apoE4 even inhibiting neurite outgrowth in some cases. Several mechanisms have been proposed. Firstly, the apoE receptor LRP1, which plays a major role in neurite outgrowth [165], was shown to be activated less effectively by apoE4 than by apoE3 [166]. Secondly, the activation of LRP1 by apoE is enhanced by the binding of apoE to heparin sulfate proteoglycan, a process that is more effective in apoE3 when compared to apoE4 [167]. In addition, the dynamics of actin polymerization, which plays an important role in neurite outgrowth and dendritic spine morphogenesis and can be stimulated via apoE receptor 2 (apoER2), is driven more effectively by apoE3 than by apoE4 [168, 169].

ApoE4 and apoE3 differ in their intracellular trafficking properties. Accordingly, following endocytosis, apoE3 readily undergoes retro-endocytosis, whereas apoE4 remains trapped in endosomes, suggesting that apoE4 clogs intracellular trafficking [170–172]. ApoE4 is associated with downregulating the levels of numerous receptors, including apoER [55, 173], as well as of growth factors and neurotransmitter receptors such as insulin [147, 148], VEGF [69], and N-methyl-D-aspartate (NMDA) receptors [33, 55], which could also play a role in impaired plasticity.

In addition to direct neuron-related mechanisms, the isoform-specific effect of apoE4 on neurite outgrowth can also be mediated by controlling the rate of microglia activation and phagocytosis [60, 123, 174, 175] as well as the activation of the complement protein C1q, which is part of the brain’s innate immune system [176].

### Summary

As demonstrated, apoE is involved in several functions, many of which are potentially relevant to AD. Studies comparing the effects of apoE3 and apoE4 highlight the expected worse functions of apoE4. However, these studies were mainly performed in animals, of short-term duration, and qualitative rather than quantitative. Therefore, it is difficult to conclude which, if any, is relevant to the human form of the disease.

The relative contribution of the mechanisms discussed above on driving the effects of apoE4 on AD pathology and their use in providing a potential therapeutic target remains to be determined. In addition, it is important to note that the link between apoE4 and AD is more pronounced in female than in male *APOE4* carriers, suggesting that specific sex-related hormones, or the lack thereof, may play a role in mediating the pathological effects of apoE4 [177, 178].

## Review of apoE4-targeted therapeutic approaches

Human studies are only able to compare differences between carriers of the various haplotypes, and thus cannot determine whether the effects of apoE4 are toxic or merely less protective. Transgenic animals either lacking apoE altogether or carrying different human haplotypes can help answer this important question. Such studies revealed that important AD pathological effects, such as the accumulation of Aβ in the brain, are significantly more pronounced in apoE4 than in apoE-deficient and apoE3 mice, suggesting that these effects are mediated via a gain of toxicity mechanism [23, 173, 179, 180]. However, other apoE4-driven phenotypes, such as astrocytic activation and synaptic loss, are similar to those observed in apoE-deficient mice [181], suggesting that they are driven by a loss of function mechanism of apoE4. Accordingly, since the levels of brain apoE4 in both AD and corresponding mice models are lower than those of apoE3 [182, 183], the effects of apoE4 could also be driven via a loss of function mechanism. It is thus possible that the effects of apoE4 in AD could be driven by multiple mechanisms, some of which could be driven by gain of toxicity and some by the loss of a protective function [117]. Since the relative contribution of such mechanisms is not known, it is not currently possible to recommend whether apoE4-related approaches should focus on counteracting apoE4 toxicity or on reversing an impaired protective mechanism. In view of this dilemma, we believe that apoE4-directed therapeutic approaches should focus primarily on the apoE4 molecule and assess both the efficacy of neutralizing the effects of apoE4, e.g., by removing apoE4 with antibodies, and of modifying the structure of the apoE4 molecule/particle to render it similar to that of apoE3, e.g., by affecting the lipidation of apoE4. The answers obtained by these complementary approaches could then pave the way for the design of an AD-directed apoE4 therapy.

Below, we focus on the aspects of apoE4 for which therapeutic approaches are being developed. A more comprehensive review of the molecular mechanisms underlying the effects of apoE4 can be found in the available reviews [17, 55, 62, 64, 184]. We first focus on the *APOE* gene and the progress achieved by clustered regularly interspaced short palindromic repeats (CRISPR) gene editing on *APOE*. Approaches targeting the apoE4 protein, focusing on attempts to counteract its effects and modify its structure, are also discussed. Subsequently, the downstream approaches that focus on the interactions of apoE4 with target proteins, such as Aβ and apoE receptors and distinct signaling cascades, are reviewed. Finally, we focus on possible therapeutic targets related to the interaction of apoE4 with the vasculature and the inflammatory systems.

### Gene editing of *APOE4* by CRISPR

The conversion of the *APOE4* gene to either *APOE3* or *APOE2* and the abolition of the concentration difference between them would lead to the ideal treatment, solving the crux of the apoE4 problem despite the incomplete understanding of the mechanisms underlying the effects of apoE4. Prior to the development of the gene-editing CRISPR technique, which enables the precise editing of genes [185], this would not have been possible. This technique is particularly suitable for the *APOE* gene, where the DNA coding for *APOE4* differs from that of the more benign isoform for AD, *APOE3*, by only one nucleotide (i.e., position 112 is arginine in *APOE4* and cysteine in *APOE3*). Ideally, the CRISPR technique could be applied for converting the *APOE4* allele to *APOE3*. However, it could also be applied in an *APOE4*-knockout paradigm which, by converting *APOE3*/*APOE4* heterozygote mice to *APOE3* homozygotes, would be expected to be protective if a toxic effect of apoE4 is assumed. CRISPR cell culture studies revealed the specific conversion of *APOE4* to an *APOE3* derivative [186], and the technique was applied to silence *APOE4* without affecting the expression of apoE3 [187]. The latter approach is expected to therapeutically counteract the presumed gain of toxicity associated with apoE4. However, successful in vivo application of CRISPR to apoE4 mice has not yet been reported. Further, it is important to note that the CRISPR technique is in its infancy and data are still emerging regarding possible off-target gene editing and mosaicism, where not all copies of the target gene are edited.

### Approaches directed at the apoE4 protein

#### Reversal of hypolipidation of apoE4

The finding that ABCA1 plays a major role in the lipidation of apoE and that apoE4 is hypolipidated led to the suggestion that the pathological effects of apoE4 are related to its extent of lipidation and that it may be possible to counteract the pathological effects of apoE4 by increasing ABCA1 activity. The expression of ABCA1 is regulated by LXR/RXR and can be activated in vivo by treatment with drugs such as bexarotene and 9-cis retinoic acid [96, 103, 188]. Treatment of apoE4 and apoE3 mice with these agents elevates the levels of ABCA1 in both groups; this was associated with a specific increase in the lipidation of brain apoE4 but with no effect on the lipidation of apoE3. The exact lipid composition of the apoE4 and apoE3 brain lipoprotein particles and the extent to which the composition is due to differences in the classes and levels of lipids associated with apoE4 remain to be determined. Additional studies utilizing apoE3 and apoE4 mice revealed that enhancing the expression of ABCA1 is associated with reversal of key apoE4 phenotypes such as the accumulation of Aβ and hyperphosphorylated tau in hippocampal neurons as well as neuronal and synaptic impairments and cognitive deficits [96, 188]. Similar results were obtained by an alternative approach in which ABCA1 was activated directly with an ABCA1 agonist [96, 103]. These animal and cellular model studies, together with genetic studies that revealed AD to be associated with polymorphism in ABCA1 [189, 190] as well as with the related transporter ABCA7 [191], suggest that apoE4 is lipidated less effectively by ABCA1 and that the resulting hypolipidated apoE4 plays an important role in mediating the pathological effects of apoE4. The mechanisms underlying the reduced lipidation of apoE4 by ABCA1 remain to be determined. However, since the levels of ABCA1 in apoE4 and apoE3 mice are comparable [102, 103], it is likely that the hypolipidation of apoE4 is due to the conformation differences between those molecules that hamper the interaction of apoE4 with ABCA1. Taken together, these findings provide strong evidence that apoE4 is hypolipidated and that this may play an important role in driving the pathological effects of apoE4. Accordingly, ABCA1 is a promising AD apoE4-related therapeutic target; this calls for further translational studies directed at the development of novel and druggable brain permeating activators of ABCA1.

#### Anti-apoE4 immunotherapy

The underlying concept of apoE4 immunotherapy is similar to that employed in Aβ and tau immunotherapy, namely to introduce or generate antibodies against these molecules in the periphery, which, following their permeation into the brain, can neutralize their target (this approach assumes a toxic effect of apoE4). Theoretically, the application of immunotherapy to apoE is faced by the problem that the levels of apoE in the periphery are approximately ten-fold higher than those in the brain [17] and that, consequently, anti-apoE antibodies could be titrated out in the periphery before reaching the brain. Contrary to this expectation, the Holtzman group has shown, utilizing amyloid precursor protein transgenic mice, that peripheral application of anti-mouse apoE can inhibit the accumulation of amyloid prior to plaque onset as well as decrease its accumulation after plaque formation [192, 193]. Although the mechanism underlying these central effects of the anti-apoE monoclonal antibodies and the reasons for them not being titrated out by peripheral apoE remain to be fully understood, these findings are of great importance and provide a proof-of-concept regarding the validity of anti-apoE4 immunotherapy as a therapeutic approach. This approach has now been extended to apoE4- and apoE3-targeted mice utilizing an antibody that reacts specifically with apoE4 [194]. This revealed that repeated intraperitoneal injection of mice with these antibodies results in their accumulation in the brain and in the formation of apoE/IgG complexes specifically in apoE4 mice. This was associated with the reversal of cognitive impairments in apoE4 mice as well as with the reversal of key AD-related and synaptic pathological effects of apoE4 [194]. These experiments, which were performed with apoE4 and apoE3 homozygous mice, are consistent with the suggestion that key pathological effects of apoE4 are mediated via a gain of toxicity mechanism.

#### ApoE4 structural correctors

ApoE4 assumes an intramolecular domain interaction that is specific to this apoE isoform and is believed to mediate its pathological effects [106]. Utilizing apoE molecules whose N- and C-terminals were fluorescently labeled coupled with a high throughput screening approach, small druggable molecules that inhibit apoE4 domain interactions and counteract the key pathological effects of apoE4 in vitro were identified [195], thus providing a proof-of-principle that correcting the pathogenic conformation of apoE4 is a viable therapeutic approach for apoE-related processes in AD.

#### ApoE degradation

ApoE4 forms an intermediate molten globule conformation that renders it less stable than apoE3 and is associated with its N- and C-terminal interaction as discussed above. This domain interaction renders apoE4 specifically susceptible to distinct proteases and leads to the generation of apoE4 carboxy terminal neurotoxic fragments [76, 77, 196, 197]. Since stress increases the neuronal production of apoE, it has been proposed that the increased production of intraneuronal apoE4 fragments under stressful conditions plays an important role in driving the pathological effects of apoE4 [76, 77, 196, 197]. Identification of the proteases involved in neuronal degradation of apoE4 and the development of inhibitors against them represent another approach for counteracting the effects of apoE4.

### Molecules interacting with apoE4 and downstream signaling

Whereas the preceding sections centered on the *APOE* gene and protein as a therapeutic target, we shall now focus on molecules with which apoE interacts and on determining the extent to which the study of such interactions can lead to identifying novel therapeutic targets. Unlike apoE4 and its gene, the focus and relative weight of an apoE4 interactor is affected by a priori assumptions such as the relative contribution of the interactions of apoE4 with Aβ and tau to the apoE4-driven pathology. Next, we highlight the therapeutic potential and limitations of the known apoE interactors.

#### ApoE-directed anti-amyloid treatment

Aβ deposition in the brains of normal controls and AD patients is higher in *APOE4* carriers [198–202] and lower in *APOE2* carriers compared with *APOE3* carriers, and it appears earlier in healthy *APOE4* carriers than in corresponding *APOE4* non-carriers [203]. Animal model studies revealed that apoE affects several key steps in the amyloid cascade, including the aggregation, deposition, and clearance of Aβ, which, like in humans, has the isoform dependency of apoE4 > apoE3 > apoE2 [52, 53]. These findings led to the suggestion that important aspects of the pathological effects of apoE4 are mediated via its interaction with Aβ and the amyloid cascade [184, 204], as well as to the development of apoE-related therapeutic strategies directed at reducing the amyloid load. This was first achieved in vitro utilizing a non-amyloidogenic Aβ-derived peptide that binds to apoE and mitigates Aβ toxicity and fibril formation [205]. More recent in vivo experiments, utilizing antibodies that recognize both human apoE4 and apoE3 and that bind preferentially to non-lipidated apoE over lipidated apoE, revealed reduced Aβ deposition in transgenic mice [206]. Reduction of amyloid pathology was also obtained using apoE antisense oligonucleotides [52]. The accepted findings that apoE4 and apoE3 bind differentially and directly to Aβ [53] have recently been challenged by Verghese et al. [207], and it is thus possible that the cross-talk between apoE4 and Aβ may be indirect and mediated via a third molecule. Although these studies clearly show that the amyloid load can be decreased by lowering the level of apoE in the brain, the apoE isoform specificity of this effect and the extent to which it can alleviate the overall isoform-specific effects of apoE4 on brain Aβ and other pathological effects of apoE4 remain to be determined.

#### ApoE receptor-related approach

Key physiological effects of apoE are mediated by the low-density lipoprotein receptor family, which includes the LDL receptor (LDLR), LRP1, the VLDLR, and the apoER2 as key players. The binding of apoE to these receptors is affected by the degree of lipidation of apoE such that non-lipidated apoE binds preferentially to LRP1 and VLDLR, whereas lipidated apoE binds more effectively to LDLR [48, 74, 208]. In addition, LRP1 and apoER2 are differentially affected by apoE4 and apoE3 [55, 209]. It is of interest to note that the receptor-mediated effects of apoE4 are associated with increased internalization and subsequent degradation of numerous receptors, including NMDA, insulin, and VEGF receptors [33, 69, 147], as well as the amyloid precursor protein and apoER2 [173, 210]. The diversity of receptors so affected by apoE4 suggests that apoE4 impairs a general receptor recycling mechanism. The pharmacology of the apoE receptors is not as rich and versatile as those of classical neurotransmitter receptors, and application of this receptor-directed pharmacology to counteract the effects of apoE4 is therefore not forthcoming. However, since one of the main effects of apoE4 is to lower the levels of apoE receptors such as apoER2 [12, 33, 69], one possible therapeutic approach could be to correct this effect by increasing the expression of apoER2 utilizing appropriate vectors.

#### ApoE mimetics

An additional therapeutic approach is the use of apoE mimetic peptides. These small peptides, which either correspond to the receptor binding domain of apoE [211–213] or a distinct apoE domain, such as amphipathic helixes domains [213], markedly reduce neurodegeneration following brain insults [212, 214–217] and protect against Aβ- and tau-driven pathology in transgenic mice and corresponding models [211–213]. The mechanism underlying the protective effects of the apoE mimetic peptides may be due to their anti-inflammatory effect. However, it should be noted that these peptides were protective following brain insults in both apoE4 and apoE3 mice [212]. Thus, assuming that these apoE mimetic peptides act and bind at the site recognized by apoE, this approach can be viewed as addressing the loss of function aspects of apoE4.

### ApoE2-focused therapeutic approach

The prevalence of apoE2 in AD subjects (2.8–4.5%) is approximately two-fold lower than in the general population and it is associated with less pronounced brain pathology than that observed in non-apoE2 AD patients [218]. *APOE2* heterozygosity is also associated with longevity [219] and reduced age-associated cognitive decline [220]. Accordingly, in neurodegenerative diseases that are associated with synaptic and neuronal loss, apoE2 is protective due to its ability to stimulate the repair of these processes. However, in age-related AMD, where excess angiogenesis in the retina is a key pathological feature, apoE2 seems to contribute to the pathology by stimulating plastic processes, which in this case mean enhanced neuro-vascularization. Several studies suggest that the brain pathological effects of apoE4 in targeted replacement mice can be counteracted by intracerebral injection of viral vectors expressing apoE2 [97, 221], suggesting a novel anti-apoE4 therapeutic approach [222]. Importantly, one of these studies also showed that apoE4 is hypolipidated relative to apoE3 and that apoE2 is hyperlipidated relative to apoE3 [97]. It is possible that apoE4 and apoE2 affect the same process, i.e., apoE lipidation, yet drive it in opposite directions. However, the possibility that apoE2 and apoE4 operate via different non-overlapping pathways with opposing physiological consequences cannot be excluded.

### ApoE4 and inflammation

Several inflammation-related targets have been proposed. These include microglia, in which the recent identification of gene expression patterns related to different stages of microglial activation presents novel targets via which microglial activation can be modulated [223, 224] and which have been shown to be effective in neurodegeneration-related models [225]. These developments and the association of apoE4 with increased neuroinflammation (see *Mitochondrial function* section above) suggest that inflammation-related treatments could be particularly effective in *APOE4* carriers. However, neuroinflammation is a double-edged sword, believed to be protective at early stages and pathological at subsequent chronic stages. The application of apoE4- and AD-related immunotherapeutic strategies is thus expected to be dependent on the stage of the inflammatory reaction at which patients are treated. Furthermore, this may vary between different brain areas. New biomarkers that identify the stage and brain location of neuroinflammation are needed to resolve this issue.

### ApoE4 and vasculature

Vascular risk factors such as hypertension, diabetes, and atherosclerosis increase the risk of AD [134, 226]. ApoE4 is associated with increased risk for vascular dementia and atherosclerosis [227, 228] as well as with impaired integrity of the vasculature and the BBB [229], suggesting that the contribution of apoE4 to AD may be driven, at least partially, by a vascular component. The identification of the molecules by which the AD-related vascular effects of apoE4 are mediated, and which could thus serve as an AD-apoE4 vascular therapeutic target, remains currently unresolved [134]. However, since important aspects of vascular diseases can be treated pharmacologically and by lifestyle modifications [230], such approaches are expected to reduce the contribution of vascular and apoE4/vascular pathology to AD.

### ApoE4 as a transcription factor

Whereas most of the suggested apoE4 pathological mechanisms are driven either extracellularly or via membrane transport and cytosolic processes, it has recently been suggested that apoE4 also undergoes nuclear translocation and that it binds specifically and with high affinity to numerous DNA sites [231]. Many of these sites are situated in promoter regions, suggesting that apoE4 could act as a transcription factor for a large number of varied genes, including autophagy and growth factor-related genes [232, 233]. Recent studies suggest that apoE4 localizes in the nucleus and that this process is related to specific proteolytic degradation of apoE4 [234]. These findings and the observation that apoE4 binds to the promoters of genes involved in a range of processes linked to aging and AD [235] led to the provocative suggestion that apoE4 may act as a transcription factor. Numerous key questions, such as how apoE escapes the endoplasmic reticulum and is trafficked to the nucleus and the impact of this mechanism relative to other pathological processes, remain to be determined. A key issue in this regard would be to determine the extent to which the pathological effects of apoE4 could be counteracted by blocking the translocation of apoE4 to the nucleus; obviously, these new observations need to be confirmed.

### Summary

**Fig. 1:**
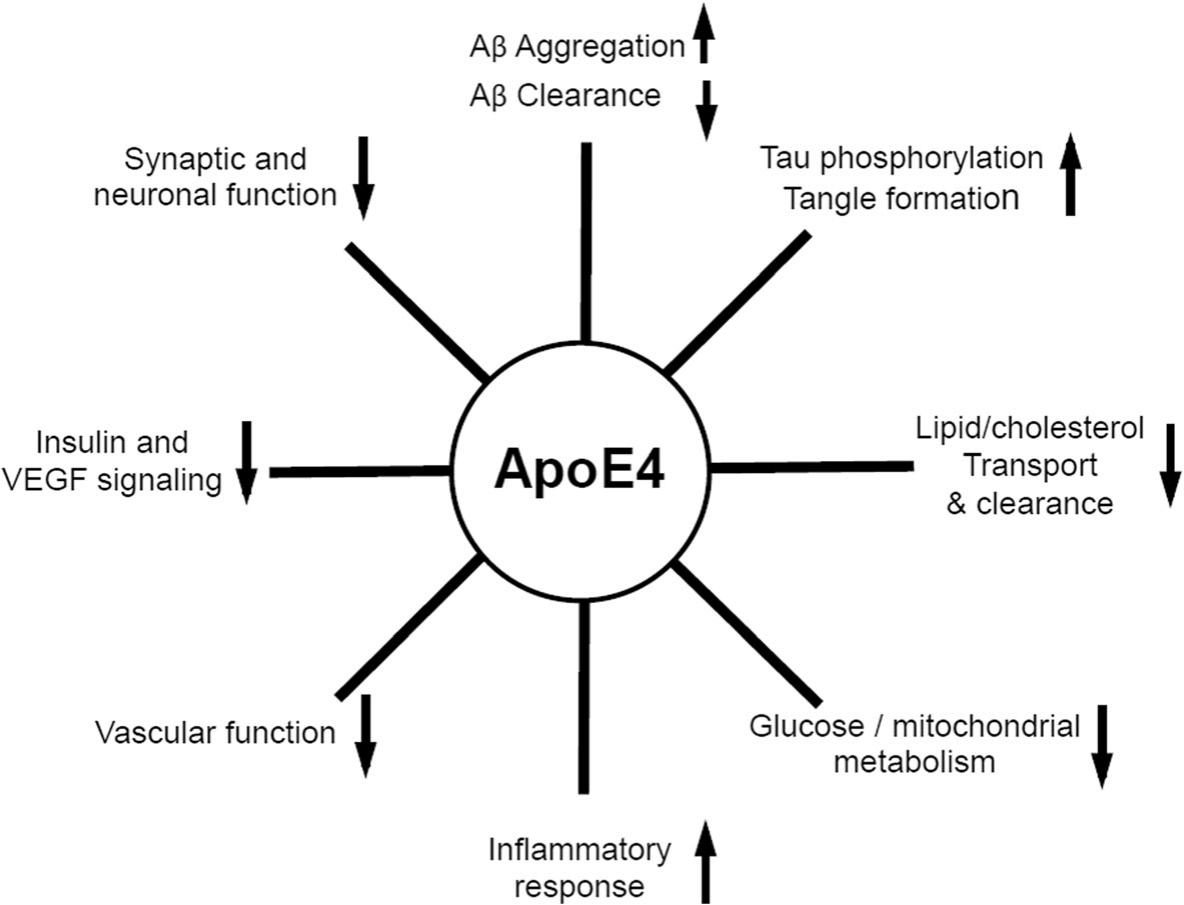
Possible therapeutic approaches targeting apoE4

We described a number of apoE4-directed approaches, ranging from the *APOE* gene to the apoE protein and its interacting molecules, in both animal and cellular model systems. These experimental approaches (Fig. 1) have been developed to counteract the pathological effects of apoE4 in mice. At present, the landscape of human apoE4-targeted therapeutic trials is bare and it is hoped that advances in animal model studies will now provide the driving force for translating these observations from the laboratory to the clinic.

ApoE4-directed therapy will first be administered to *APOE4* carriers who express early signs of the disease, such as mild cognitive impairments, and early imaging changes such as hippocampal atrophy. Following a successful pursuit of this protocol, the effectivity of this treatment will be assessed when provided at more advanced disease stages. Prophylactic administration to *APOE4* carriers could also be considered depending on the drug safety profile.

**Fig. 2:**
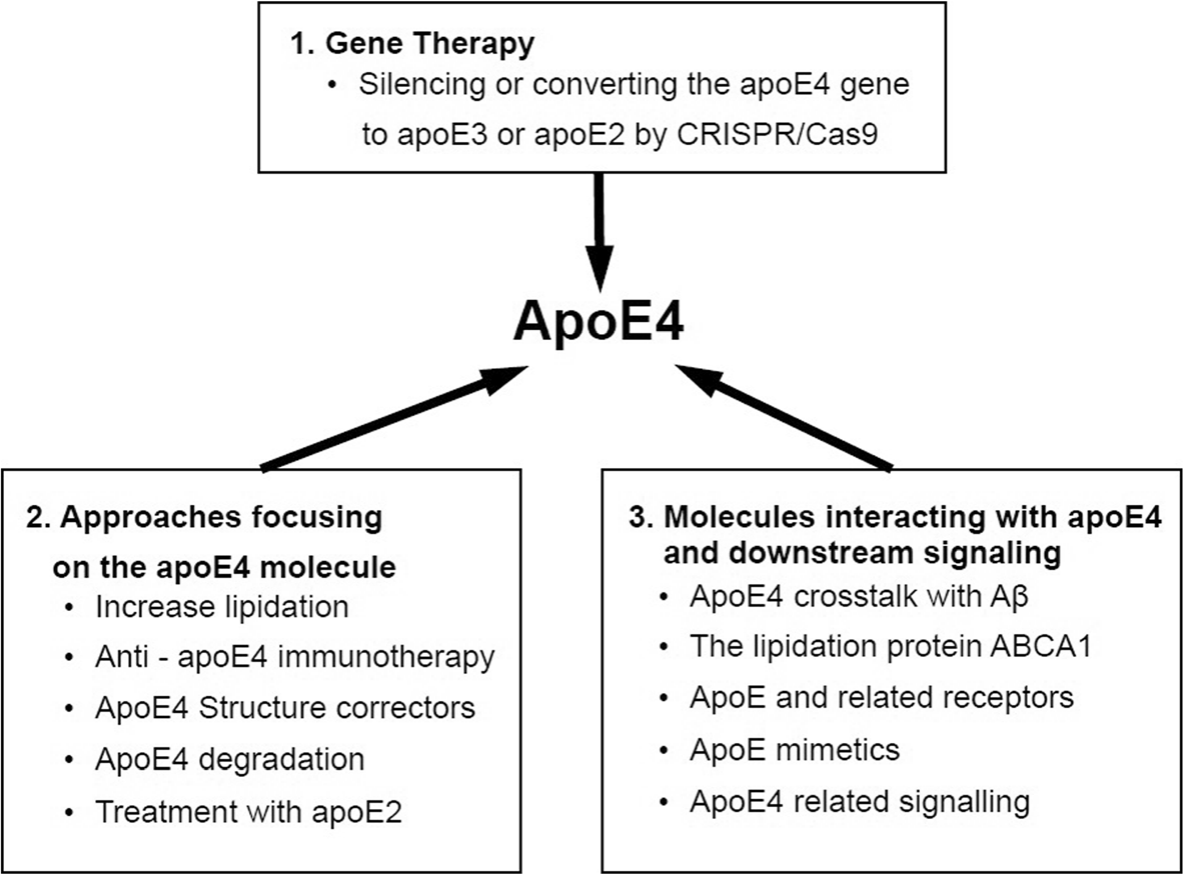
Schematic presentation of the apoE4-driven mechanisms involved in AD pathology

A schematic summary of all the proposed apoE-driven pathological mechanisms is presented in Fig. 2.

## Conclusion

The upcoming development of specific therapies related to apoE4 raises several questions. First, will this therapy be curative rather than preventive? In other words, is it possible that AD patients who carry the *APOE*4 allele will improve when treated with the new therapy? It is possible that such treatment would slow the rate of decline in *APOE4* carriers, yet it is likely that it would not entirely halter the neurodegenerative process. The greatest potential of anti-apoE4 therapy therefore lies in delaying the onset and progression of dementia, rather than curing the disease. If such a therapy were to be initiated in non-demented individuals carrying the *APOE4* allele, disease onset could be delayed by at least approximately 7 years per *APOE4* allele; this intriguing possibility then poses the question of when therapy should commence. Another likely benefit of anti-apoE4 therapy is a reduction in the associated morbidities, e.g., cardiovascular disease, particularly coronary artery disease and impaired repair following head trauma, which are more common among *APOE4* carriers [236].

## Abbreviations

AD: Alzheimer’s disease
AMD: age-related macular degeneration
apoE: apolipoprotein E
apoER: apolipoprotein E receptor
Aβ: amyloid beta
BBB: blood–brain barrier
CAA: cerebral amyloid angiopathy
CRISPR: clustered regularly interspaced short palindromic repeats
CSF: cerebrospinal fluid
DHA: docosoahexaenoic acid
DLB: dementia with Lewy bodies
EOAD: early-onset Alzheimer’s disease
LDL: low-density lipoprotein
LDLR: LDL receptor
LOAD: late-onset Alzheimer’s disease
LRP1: LDL receptor-related protein 1
NMDA: N-methyl-D-aspartate
NSAID: non-steroidal anti-inflammatory drug
VEGF: vascular endothelial growth factor
VLDLR: very low-density lipoprotein receptor
